# Systematic investigation of transcription factors critical in the protection against cerebral ischemia by Danhong injection

**DOI:** 10.1038/srep29823

**Published:** 2016-07-19

**Authors:** Junying Wei, Yanqiong Zhang, Qiang Jia, Mingwei Liu, Defeng Li, Yi Zhang, Lei Song, Yanzhen Hu, Minghua Xian, Hongjun Yang, Chen Ding, Luqi Huang

**Affiliations:** 1 Institute of Chinese Materia Medica, China Academy of Chinese Medical Sciences, Beijing 100700, China; 2 State Key Laboratory of Proteomics, Beijing Proteome Research Center, Beijing Institute of Radiation Medicine, Beijing 102206, China; 3 State Key Laboratory of Genetic Engineering and Collaborative Innovation Center for Genetics and Development, School of Life Sciences, Institute of Biomedical Sciences, Fudan University, Shanghai 200433, China; 4 State Key Laboratory Breeding Base of Dao-di Herbs, National Resource Center for Chinese Materia Medica, China Academy of Chinese Medical Sciences, Beijing 100700, China

## Abstract

Systematic investigations of complex pathological cascades during ischemic brain injury help to elucidate novel therapeutic targets against cerebral ischemia. Although some transcription factors (TFs) involved in cerebral ischemia, systematic surveys of their changes during ischemic brain injury have not been reported. Moreover, some multi-target agents effectively protected against ischemic stroke, but their mechanisms, especially the targets of TFs, are still unclear. Therefore, a comprehensive approach by integrating network pharmacology strategy and a new concatenated tandem array of consensus transcription factor response elements method to systematically investigate the target TFs critical in the protection against cerebral ischemia by a medication was first reported, and then applied to a multi-target drug, Danhong injection (DHI). High-throughput nature and depth of coverage, as well as high quantitative accuracy of the developed approach, make it more suitable for analyzing such multi-target agents. Results indicated that pre-B-cell leukemia transcription factor 1 and cyclic AMP-dependent transcription factor 1, along with six other TFs, are putative target TFs for DHI-mediated protection against cerebral ischemia. This study provides, for the first time, a systematic investigation of the target TFs critical to DHI-mediated protection against cerebral ischemia, as well as reveals more potential therapeutic targets for ischemic stroke.

Cerebral ischemia or stroke is a serious neurological disease, which can lead to broad cerebral injury and result in high disability and mortality rates in many countries^1^. Therefore, systematic investigations of the complex pathological cascades during ischemic brain injury help to develop effective treatments and elucidate novel therapeutic targets against cerebral ischemia^2^. It is known that the pathological events during ischemic stroke include inflammation, excitotoxicity, mitochondrial depolarization, oxidative stress, and apoptosis^3^. Among the signaling molecules involved in the pathogenesis of ischemic stroke, transcription factors (TFs), as key regulators of many cellular processes^4^, may play a vital role and act as potential therapeutic targets^5^,^6^,^7^,^8^,^9^,^10^,^11^,^12^. For example, aryl hydrocarbon receptor (AhR)^13^, was found to be an important mediator of acute ischemic damage during middle cerebral artery occlusion (MCAO)^2^. Collaboration between hypoxia inducible factor-1α (HIF-1α) and Notch-1 promotes neuronal cell death in ischemic stroke^5^. Recently, high-mobility group I-Y (HMGIY) was reported to be involved in cerebral ischemia by modulating the expression of angiogenic proteins^6^. Although several investigations on the role of some TFs in cerebral ischemia had been performed, systematic surveys of the changes of multiple TFs during the ischemic brain injury have not been reported, and further work is actually needed to look for more potential therapeutic targets.

A computational approach, such as a network pharmacology-based^14^,^15^,^16^ or microarray analysis-based^17^ approach, is suitable for comprehensively investigating the roles of TFs in cerebral ischemia. By employing systems biology and network analysis^15^, we could computationally and systematically understand how many and which TFs might be involved in cerebral ischemia. Although the approach is promising, many techniques, such as network search algorithms and methods for predicting the biological profiles, will still need to be refined^14^. Moreover, the obtained results still need further strict experimental validations.

Large-scale quantitative profiling of TFs during ischemic brain injury can be another good choice to comprehensively investigate their roles in cerebral ischemia. However, due to the low abundance of TFs^18^, large-scale profiling still remains a challenge, let alone quantitative analysis. Moreover, the most meaningful analysis of TFs is to monitor their binding activities to specific DNA sequences when they are perturbed because it is a delineation of signal transduction pathways. To meet the requirement of multiple analyses of activated TFs, a synthetic DNA containing a concatenated tandem array of consensus transcription factor response elements (catTFREs) for most known TF families was successfully used to large-scale detection of activated TFs^19^. Most importantly, this approach could quantitatively measure changes in TF activation, which is promising for systematically elucidating the roles of TFs in cerebral ischemia and revealing the corresponding therapeutic drug targets.

In this study, an approach by integrating network pharmacology strategy and the catTFREs method to systematically investigate the target TFs critical in the protection against cerebral ischemia by a medication was first reported, and then applied to a multi-target conventional drug for coronary heart disease and cerebral ischemia, Danhong injection (DHI)^20^,^21^,^22^,^23^. Although DHI, as a Chinese Materia Medica standardized product extracted from *Radix Salviae miltiorrhizae* and *Flos Carthami tinctorii*, has been shown to be effective in protecting against ischemic stroke, its pharmacological mechanisms, especially the putative multi-targets of TFs, are still unclear. This study provides the first systematic investigation of the TFs critical to DHI-mediated protection against cerebral ischemia, as well as reveals more potential therapeutic targets for ischemic stroke.

## Experimental Section

### Materials and Reagents

Concatenated tandem array of consensus transcription factor response element (catTFRE) DNA was synthesized by Genscript (Piscataway, NJ, USA). Biotinylated catTFRE primers were synthesized by Sigma (St. Louis, MO USA). Dynabeads (M-280 streptavidin) were purchased from Invitrogen. Sequencing grade porcine trypsin was obtained from Promega (Madison, WI, USA). Nuclear extract prep kits were purchased from Thermo Fisher. Danhong injection (DHI) was obtained from Shandong Danhong Pharmaceutical Co., Ltd (Shandong, China). All other chemicals were of analytical grade reagent. Deionized water (R > 18.2 MΩ) used for all experiments was purified by using Millipore purification system (Billerica, MA, USA).

### Animal Study

Male C57BL/6 mice (Charles River Laboratories), six-eight weeks of age, were treated by intraluminal occlusion using monofilament for preparation of the permanent middle cerebral artery occlusion (MCAO) model^24^,^25^. Briefly, mice were first anesthetized with 5% chloral hydrate. Then a short vertical skin incision was made between the left eye and ear. Common carotid artery (CCA), internal carotid artery (ICA) and external carotid artery (ECA) were isolated. A silicone coated nylon suture was then introduced through the ICA and ECA stump. Finally, a silicone coated nylon filament was inserted into CCA by pushing it 9 mm distal from the carotid bifurcation, and occluded the middle cerebral artery. Interruption of blood flow at the occlusion site was continuously monitored by laser-Doppler flowmetry to ensure the adequacy of MCAO. The whole operation process were completed within 15 minutes, and the rectal temperature of the mouse model was maintained at 37 ± 0.5 °C throughout the surgical procedure. Sham mice underwent the same procedures as above mentioned with the exception of insertion of the nylon filament into CCA. All animal experiments were approved by the Committee on Animal Care and Use of Institute of Chinese Materia Medica, China Academy of Chinese Medical Sciences, and carried out in accordance with the approved guidelines.

Based on the literatures about the processes after cerebral ischemia^26^,^27^,^28^, six hours later after MCAO-operation, the neurologic function was evaluated blindly by Longa’s Neurological Severity Score^24^. Then mice were euthanized with a lethal dose of isoflurane. Seven coronal sections of the brain (1 mm thickness) were immediately cut, then the slices were immediately stained with 0.5% 2,3,5-triphenyltetrazolium chloride (Sigma, St. Louis, MO USA) for 15 minutes at 37 °C and numeric images were captured for quantification of Infarct Volume.

To evaluate the protection effect of DHI against cerebral ischemia, each MCAO mouse was administered 5 mL/kg/time DHI immediately after MCAO-operated by intraperitoneal injection (i.p.), then twice per day, respectively in the morning and evening, for eight consecutive days. For control, each sham mouse without MCAO was also administered 5 mL/kg/time DHI, and the usage is same as above described.

### catTFRE Pull-Down and Digestion with Trypsin

Nuclear extracts of the mouse brain were prepared by using nuclear extract prep kits (Thermo Fisher) according to the protocols of manuals. Biotinylated DNA was pre-immobilized on Dynabeads and then mixed with nuclear extract. The mixture was supplemented with EDTA/EGTA to a final concentration of 1 mM and adjusted with NaCl to 200–250 mM total salt concentration. The solution was then incubated at 4 °C for 2 h. The supernatant was discarded and Dynabeads were washed with NETN [100 mM NaCl, 20 mM Tris-HCl, 0.5 mM EDTA, and 0.5% (vol/vol) Nonidet P-40] twice followed by PBS solution twice. Beads were digested overnight with trypsin.

### LC-ESI-MS/MS Measurement and Protein Quantifications

Tryptic peptides were separated on a C18 column (75 μm inner-diameter, 360 μm outer-diameter × 10 cm, 3 μm C18) with a flow rate of 350 nL/min, and were analyzed by LTQ-Orbitrap Velos (Thermo). The MS conditions were as the followings: Nano-spray ion source was used. A spray voltage of 1800 V was applied, with no sheath gas flow and with the ion transfer tube at 350 °C. The mass spectrometer was programmed to acquire in a data dependent mode. The survey scan was from m/z 375 to 1600 with resolution 60,000 at m/z 400. The 50 most intense peaks with charge state 2 and above were acquired with collision induced dissociation with normalized collision energy of 35%, activation time of 5 ms, one microscan and the intensity threshold was set at 500. The MS2 spectra were acquired in the LTQ normal scan mode. Proteins were identified by Proteome Discovery version 1.3 using MASCOT search engine with percolator against the mouse RefSeq protein database (updated on 11-17-2014). The mass tolerance was set to be 20 ppm for precursor. As for the tolerance of product ions, Velos was set as 0.5 Da. Oxidation (Met), Acetyl (N terminus) was chosen as variable modifications; carbamidomethyl (Cys) was chosen as a fixed modification; and one missed cleavage on trypsin was allowed. Both peptide and protein level false discovery rates (FDRs) were controlled lower than 1%. Transcription factors (TFs) were assigned based on TFClass^29^.

Intensity-based absolute quantification (iBAQ)-based protein quantifications^30^ were performed by an in-house software. Briefly, the iBAQ intensities were obtained by dividing the protein intensities by the number of theoretically peptides which were calculated by *in silico* protein digestion with a PERL script, and all fully tryptic peptides between 6 and 30 amino acids were counted while missed cleavages were neglected.

### Structural Information of Chemical Components of Each Herb in DHI

Structural information (*.mol or *.sdf files) of the chemical components of each herb in DHI were obtained from TCM Database@Taiwan^31^ (http://tcm.cmu.edu.tw/, Updated in Jun 28, 2012), which is currently the largest non-commercial TCM database worldwide. Totally, structural information of 101 compounds for *Radix Salviae miltiorrhizae* and 22 compounds for *Flos Carthami tinctorii* were collected from this database.

### Known Therapeutic Targets for Ischemic Stroke

Known therapeutic targets for ischemic stroke were collected from DrugBank database^32^ (http://www.drugbank.ca/, version: 3.0) and the Online Mendelian Inheritance in Man (OMIM) database^33^ (http://www.omim.org/, Last updated: October 31, 2013). After deleting redundancy, 62 known therapeutic targets for the treatment of ischemic stroke were collected in this study. Please see detailed information in Supplementary Table S1.

### Protein-protein Interaction (PPI) Data

Two existing PPI databases, including Reactome^34^ (http://www.reactome.org/, Version 37) and String^35^ (http://www.string-db.org/, Version 9.1) were used for the collection of PPI data.

### Prediction of Putative Targets for DHI

Putative targets of the chemical components contained in *Radix Salviae miltiorrhizae* and *Flos Carthami tinctorii* were predicted by MetaDrug from GeneGo, Inc. MetaDrug predicts the putative targets for certain chemical components using three methods: (1) Based on the MetaBase database, which contains compound-protein interactions; (2) Based on QSAR predictions of protein target affinity from the included models that define a limited number of potential targets for novel molecules and/or their metabolites submitted for analysis; (3) Based on a similarity search for the structure and its major metabolites against the database of existing structures and their targets. Putative targets for certain compounds are inferred through structurally similar compounds in the database (GeneGo, personal communication).

### Network Construction

Chemical components contained in DHI, the corresponding putative targets, known therapeutic targets for ischemic stroke and the differentially expressed TFs in MCAO models and controls were used to construct two interaction networks: putative targets-known therapeutic targets-TFs network and chemical components-major putative target-major TFs network. Navigator software (Version 2.2.1) and Cytoscape (Version 2.8.1) were utilized to visualize the networks.

### Network Topological Analysis

Four topological features for each node in the network, including ‘Degree’, ‘Betweenness’, ‘Closeness’ and ‘K value’ were calculated to evaluate the topological importance of DHI putative targets and the differentially expressed TFs in MCAO models and controls. For each node “i” in these two networks, “Degree” was defined as the number of links to node i; “Betweenness” was defined as the number of edges running through node i; “Closeness” was defined as the inverse of the sum of node i distances to all other nodes. “Degree”, “Betweenness” and “Closeness” correlate with a protein’s topological importance in the PPI network^36^. In addition, the K-core analysis is an iterative process in which nodes were removed from the network in order of the least-connected^37^. “K value” is used to measure the centrality of node i. To identify the major nodes in the network, we chose those the values of the four features were higher than the corresponding median values.

### Electrophoretic Mobility Shift Assay (EMSA)

Nuclear extracts of the mouse brain were prepared by using nuclear extract prep kits (Thermo Fisher) as above mentioned. EMSA was performed by using the LightShift chemiluminescent EMSA kit (Pierce Biotechnology, Rockford IL) according to the protocols of manuals. The sense strand sequences of the oligonucleotides for the EMSAs are as follows: ATF1, 5′-ATGACGTCAATGACGTCAATGACGTCA-3′, Mut-DNA of ATF1, 5′-ACGATGTCAACGATGTCAACGATGTCA-3′; PBX1, 5′-ATCAATCAAATCAATCAAATCAATCAA-3′, Mut-DNA of PBX1, 5′-ACATATAACACATATAACACATATAAC-3′. Nuclear protein (6 μg) was mixed with 10 × binding buffer (Thermo Fisher), 2.5% glycerol, 5 mM MgCl_2_, 50 ng poly(dI-dC), 0.05% NP-40, and incubated with 10 fmol of biotin-labeled DNA probe for 15 min at room temperature. DNA probe/protein complexes were then separated by 5% native polyacrylamide gel at 100 V for 60 min, followed by transferred to a nylon membrane (GE Healthcare), and visualized by chemiluminescence according to the manufacturer’s instructions (GE ImageQuant LAS 4000mini).

### qPCR assay

qPCR was performed with primers listed in Supporting Information on an ABI 7900 Instrument (Applied Biosystems). The SYBR FAST qPCR Master Mix (Applied Biosystems) was used. The value of each mRNA expression was normalized by GAPDH mRNA expression. The change in the Ct (ΔCt) was calculated as ΔCt = (Ct of target gene)−(Ct of GAPDH). The ratio was calculated as 2^−ΔCt^. Then relative differences of gene expression among groups were expressed as relatively changes, setting the values of sham mice as one. The assays were carried out in triplicate and the results were analyzed by one-way analysis of variance, and significant at P < 0.05.

## Results and Discussion

### Transcription factors (TFs) changes after ischemic brain injury in mouse MCAO models

First, we measured the changes in TF activation in the brain after ischemic injury using the catTFREs method and the intensity-based absolute quantification (iBAQ)-based quantification approach, which had been demonstrated to be accurate in protein quantification^30^,^38^,^39^. Based on the literature regarding the time window of therapeutic opportunity after cerebral ischemia^26^,^27^,^28^, the activated TFs in ischemic brain from the mouse MCAO model were analyzed six hours after MCAO operation. Meanwhile, to perform accurate quantification analyses, the iBAQ value of a TF was normalized by the total iBAQ value for all of the identified proteins to avoid possible experimental variations^39^,^40^. As shown in Fig. S1, six hours after MCAO operation, both Longa’s Neurological Severity Score (Fig. S1a) and triphenyltetrazolium chloride (TTC) staining (Fig. S1b) of brain tissue slices revealed severe ischemic injury. When analyzing the corresponding activated TFs in pooled samples of three ischemic brains and three sham controls, a total of 250 activated TFs were identified as a result (Table S2). According to the literature^41^, there are approximately 200 TFs expressed in each tissue. Thus the depth of coverage for activated TFs detection by the catTFREs method makes it as a promising approach to systematically elucidate the roles of TFs in cerebral ischemia and to reveal novel potential therapeutic targets against cerebral ischemia.

**Figure 1:**
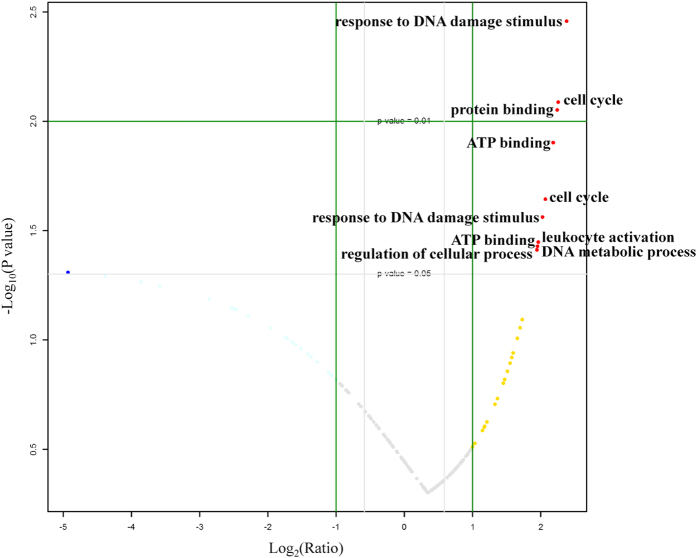
Activated TFs’ change at six hours later after MCAO-operation in mice MCAO models and the biological process or molecular function of some TFs with significantly different activation levels (p < 0.05).

Among the 250 identified activated TFs, 47 TFs were expressed only in sham controls, while 19 were expressed only in ischemic brains (Table S2). Moreover, compared with the sham control group, 91 TFs (91/184, almost 50%) in ischemic brains showed a fold change of >1.3 and 41 showed a fold change of <0.7 (Table S2), indicating that many TFs may be activated and involved in cerebral ischemia. Fold changes of the quantified activated TFs in ischemic brains compared with the sham control are shown as in Fig. 1. Some TFs had significantly different activation levels (p < 0.05). Using the GOfact tool^42^, their biological process or molecular function was investigated, which was found to function in various cellular protein processes, such as the response to DNA damage stimulus, cell cycle, protein or ATP binding, leukocyte activation, and others (Fig. 1). Moreover, some putative TF targets reported in the literature^2^,^6^ were clearly changed. For example, aryl hydrocarbon receptor (AhR) nuclear translocator (ARNT, AhR partner)^2^ and high-mobility group I-Y^6^ were upregulated 3.3-fold and 4.7-fold, respectively (Table S2), which indicates that our approach can accurately and quantitatively measure changes in TF activation, which can facilitate finding novel potential therapeutic targets.

From the fold changes of the quantified activated TFs in ischemic brains compared with the controls (Fig. 1), we also found that many TFs did not have significantly different activation levels (>4-fold, p < 0.05). In fact, using the catTFREs method and iBAQ-based quantification, what we found is the binding activities of TFs to specific DNA sequences when they are perturbed. Additionally, in some cases, the fold change of the TF binding activities are not so high^2^,^43^. Thus, to find more novel potential therapeutic targets, we adopted the criterion of fold changes >1.3 or <0.7, which covered almost 75% of identified activated TFs in both ischemic brains and controls, to select TFs that had altered activation levels during ischemic brain injury for the computational prediction of potential targets. Detailed information is listed in Table S2.

### Prediction of TFs critical to the therapeutic effects of DHI on ischemic stroke

Next, using network pharmacology strategies, TFs critical to DHI-mediated protection against cerebral ischemia were computationally predicted. First, a total of 660 putative targets were predicted for 57 chemical components contained in DHI. Detailed information regarding the putative targets and corresponding chemical components contained in DHI is listed in Table S3. Next, to shed light on the relationship between the 151 differentially expressed activated TFs during ischemic brain injury and the therapeutic effect of DHI on ischemic stroke (listed in Table S2), we constructed the TFs-putative targets-known therapeutic targets network.

**Figure 2:**
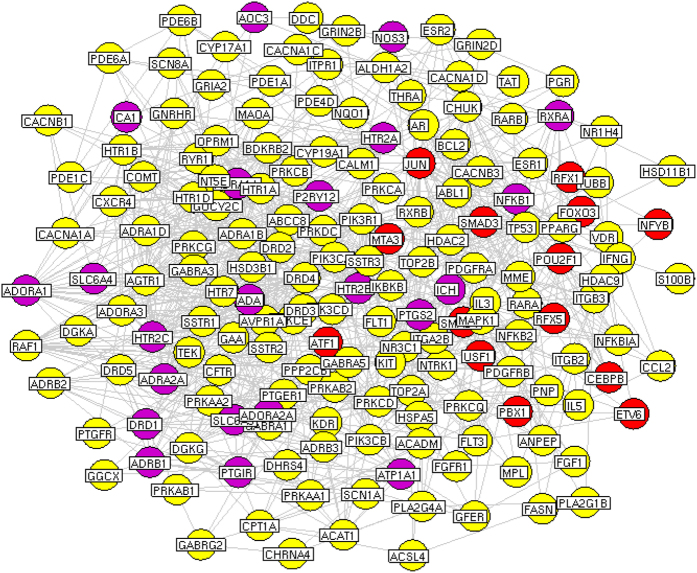
Network of interactions among 175 major nodes. Red round nodes refer to 14 major TFs; Yellow refer to the putative targets of DHI; Purple refer to known therapeutic targets for stroke/the putative targets of DHI.

The network consists of 553 nodes (including 151 TFs that were differentially expressed during the ischemic brain injury, 62 known therapeutic targets for ischemic stroke and 340 putative targets for DHI) and 2204 edges. Four topological features, ‘Degree’, ‘Betweenness’, ‘Closeness’ and ‘K value’ (defined in EXPERIMENTAL SECTION), were chosen to identify major putative targets and major differentially expressed TFs. In total, we identified 175 major nodes, the ‘Degree’, ‘Betweenness’, ‘Closeness’ and ‘K value’ of which are all larger than the corresponding median values. Among these major nodes, there are 140 major putative targets and 14 major differentially expressed TFs. The interaction network of 175 major nodes is shown in Fig. 2 and Table S4. Please see detailed information on topological features of the major nodes in the TFs-putative targets-known therapeutic targets network in Table S5.

### Evaluation of the pharmacological effects of DHI on MCAO mice

**Figure 3:**
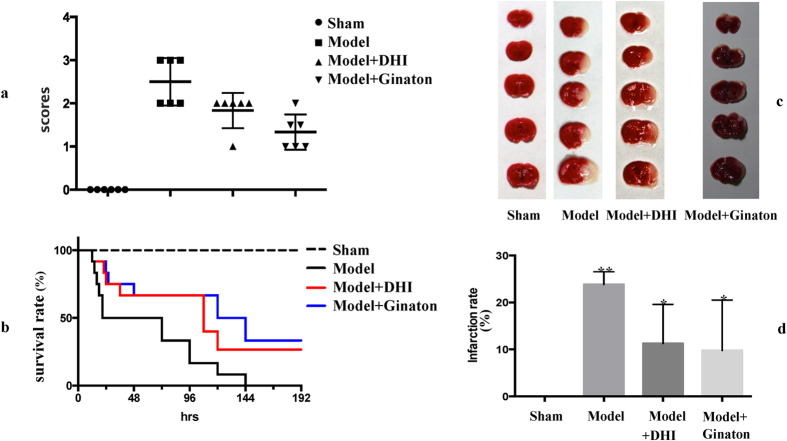
Drug effect of DHI on MCAO mice. (**a**) Longa’s Neurological Severity Score; (**b**) Eight-day (192 h) survival rate test; (**c**) TTC staining of the brains; (**d**) Infarction rate.

To experimentally validate the predicted target TFs crucial to the DHI-mediated protection against cerebral ischemia, first, the pharmacological effects of DHI on MCAO mice was evaluated. As shown in Fig. 3a, six hours after MCAO operation, Longa’s Neurological Severity Score from model group revealed remarkable ischemic injury, while both DHI and the positive control, ginaton, can observably decrease the scores, indicating improved neurological function in MCAO mice. Moreover, the eight-day (192 h) survival rate test (Fig. 3b) indicated that approximately 50% of MCAO mice without any treatment died within 24 h, and none of the other mice survived longer than 144 h. Both DHI and positive control ginaton can remarkably prolong the survival rate of animals. Even at 192 h after MCAO operation, almost 30% of MCAO mice survived, indicating that DHI is effective in protecting against ischemic stroke.

To further investigate the pharmacological effects of DHI on MCAO mice, another group of mice that received DHI were euthanized six hours after MCAO operation and their brains were stained with TTC. As shown in Fig. 3c, TTC staining of the brains from these mice treated with DHI showed a lower degree of ischemic injury than the MCAO mice that did not receive any treatment. Additionally, the corresponding infarction rate (Fig. 3d) also indicated that both DHI and positive control ginaton have significant protective effects against ischemic injury. Thus, all of these experimental results affirmed a reliable protective effect of DHI on ischemic stroke. Moreover, such protective effect by DHI and the corresponding mechanism had also been reported by many researchers^22^,^23^,^44^, such as antifibrinolytic and antioxidant^22^, activating Nrf2 signaling pathway^23^ and inhibiting platelet activation^44^. And in this study, we focused on the critical TFs to this protection.

### Further verification of TFs critical to the protective effects of DHI against cerebral ischemia

**Figure 4:**
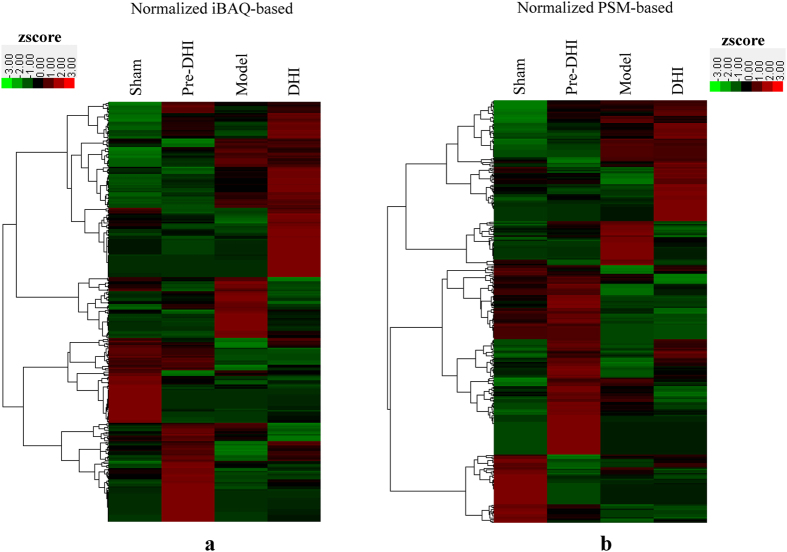
Hierarchical clustering of the quantitative information from activated TFs between MCAO and sham mice with and without DHI administration. (**a**) normalized iBAQ-based; (**b**) normalized PSM-based. A zscore transformation was used. Pre-DHI or DHI group means sham or MCAO mice with DHI administration.

Based on the reliable protective effect of DHI against cerebral ischemia, we further experimentally verified the TFs that were critical to this protection by applying the catTFREs method and the accurate iBAQ-based quantitative approach to comprehensively measure the corresponding change in TF activation in pooled samples from three ischemic brains after DHI treatment. Meanwhile, to further confirm these TFs as critical, we conducted a parallel test by administering the same amount of DHI to sham mice without MCAO, followed by a high-throughput analysis of the corresponding changes in TF activation using the same method mentioned above. Moreover, to attain accurate quantitative analysis, peptide-spectrum match (PSM)^45^ was selected as a complementary quantitative parameter. As a result, six hours after MCAO operation, 297 activated TFs and the changes between MCAO mice with or without DHI treatment and sham mice with or without DHI treatment are shown in Table S6. Then, after the iBAQ-based and PSM-based quantitative information of these 297 activated TFs was filtered by hierarchical clustering^46^ (Fig. 4), 16 remarkably changed TFs confirmed by both quantitative approaches were considered as candidate critical TFs to DHI-mediated protection against cerebral ischemia and also listed in Table S6.

From the results of the computational prediction and the experimental verification of TFs critical to DHI-mediated protection against cerebral ischemia, we found that pre-B-cell leukemia transcription factor 1 (PBX1) and cyclic AMP-dependent transcription factor 1 (ATF1) were authenticated by both approaches. PBX1 binds the sequence 5′-ATCAATCAA-3′ and may act as a transcriptional activator of platelet factor 4 (PF4) in complex with homeobox protein Meis1 (MEIS1)^47^, which plays an important role in megakaryocytic gene expression^48^. In addition, as an important protein colocalized with MEIS2 in developing striatal neurons, PBX had been reported to be expressed in stroke-generated new cells in the striatum^49^. ATF1 binds the cAMP response element (CRE) (consensus: 5′-GTGACGT[AC][AG]-3′), which is present in many cellular and viral promoters^47^. It mediates PKA-induced stimulation of CRE-reporter genes, regulates the expression of ferritin heavy chain and other antioxidant detoxification genes^50^, and triggers cell proliferation and transformation^47^. In our computational prediction results, PBX1 and ATF1 were indicated as major putative targets, and in our experimental verification results, their transcription activities were remarkably changed whether between MCAO mice with and without DHI treatment or between sham mice with and without DHI treatment. Thus, we concluded that PBX1 and ATF1 are putative target TFs for DHI-mediated protection against cerebral ischemia.

**Figure 5:**
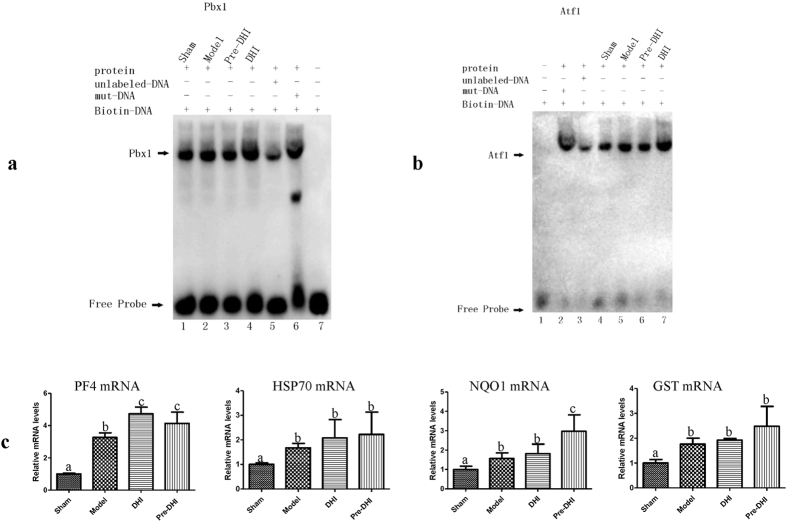
Further verification of the TF activities of PBX1 and ATF1. (**a**) EMSA result of PBX1. Pre-DHI or DHI group means sham or MCAO mice with DHI administration; (**b**) EMSA result of ATF1; (**c**) qPCR assay of downstream effectors changes after MCAO and DHI administration. Significant differences (p < 0.05) among (**a–c**) are determined by ANOVA.

To further verify the TF activities of PBX1 and ATF1, the electrophoretic mobility shift assay (EMSA) experiments were conducted. Both PBX1 and ATF1 showed a stronger binding in MCAO mice with DHI treatment than in the ones without DHI treatment. And the same result was also obtained between sham mice with and without DHI treatment (Fig. 5a,b). Furthermore, downstream effectors changes after MCAO and DHI administration were also measured by qPCR. As shown in Fig. 5c, PF4, which activated by PBX1^48^, showed a change in its mRNA expression after MCAO and DHI administration. And the downstream effectors of ATF1, such as heat shock protein 70 (HSP70)^51^, NADPH quinone oxidoreductase 1 (NQO1) and glutathione S-transferase (GST)^50^, also showed obvious changes. Thus all the experimental results further proved the target transcription factors for the DHI therapeutic effects.

Moreover, from our experimental verification results, we also found out that, in addition to PBX1 and ATF1, six other TFs, nuclear transcription factor Y subunit gamma (NFYC), POU domain, class 3, transcription factor 1 (POU3F1), POU3F2, POU3F3, DNA-binding protein SATB1 (SATB1) and SATB2, may also be critical target TFs for DHI-mediated protection against cerebral ischemia. NFYC is the component of the sequence-specific heterotrimeric TF (NF-Y) that specifically recognizes a 5′-CCAAT-3′ box motif found in the promoters of its target genes^47^. POU3F1 binds to the octamer motif (5′-ATTTGCAT-3′) and is thought to be involved in early embryogenesis and neurogenesis^47^. POU3F2 binds preferentially to the recognition sequence, which consists of two distinct half-sites, (‘GCAT’) and (‘TAAT’). POU3F3 acts synergistically with TFs SOX11 and SOX4 and plays a role in neuronal development^52^. SATB1 binds to DNA at special AT-rich sequences and plays a role in chromatin organization and nuclear architecture during apoptosis^47^. SATB2 recognizes the sugar-phosphate structure of double-stranded DNA and controls nuclear gene expression. Preliminary results already indicated that the upregulation of NFYC contributes to neuronal apoptosis via proapoptotic protein Bim in rats’ brain hippocampus after MCAO^53^. Additionally, POU3F3 was moderately induced in the CA1 subregion of hippocampal formation by 3–6 h after ischemia, and its expression is regulated by the neuronal activity and altered after brain ischemia^52^. Moreover, SATB2 was reported to be positively regulated by POU3F3/POU3F2^54^, and in our experimental verification results, it also appeared that the expression patterns of SATB2 and POU3F3 were similar to each other. More detailed research is needed to investigate the functions and mechanisms of these 6 TFs in the pathophysiology of MCAO and the DHI-mediated protection against cerebral ischemia. However, considering their remarkably altered transcription activity levels between MCAO and sham mice with and without DHI treatment, we still infer that they may be critical to the protection against cerebral ischemia by DHI.

**Figure 6:**
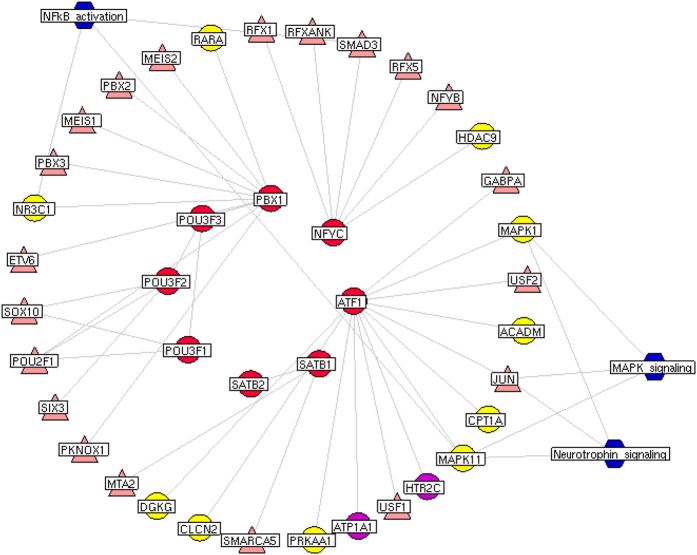
Network of interactions among eight major TFs, putative targets of DHI, known therapeutic targets for stroke and other differentially expressed TFs, and the stroke-related pathways involved by these genes. Red round nodes refer to the eight major TFs; Yellow refer to the putative targets of DHI; Purple refer to known therapeutic targets for stroke/the putative targets of DHI; Pink refer to other differentially expressed TFs; Blue refer to three crucial stroke-related pathways.

To further clarify the relationships of eight major target TFs with major putative targets of DHI and known therapeutic targets for ischemic stroke, we constructed a network of interactions among these genes as shown in Fig. 6. Among eight major target TFs, PBX1, NFYC and ATF1 all had direct interactions with putative targets of DHI. Particularly, the interaction partners of ATF1, HTR2C and ATP1A1 were not only the putative targets of DHI but also known therapeutic targets for ischemic stroke. According to the pathway enrichment analysis, we found that the interaction partners of eight major target TFs were more frequently involved in MAPK signaling (MAPK1, MAPK11 and JUN), neurotrophin signaling (MAPK1, MAPK11 and JUN) and NF-kB activation signaling (SMAD3, MAPK11 and NR3C1) pathways, which all play crucial roles in the pathological processes of ischemic stroke^55^,^56^,^57^,^58^.

**Figure 7:**
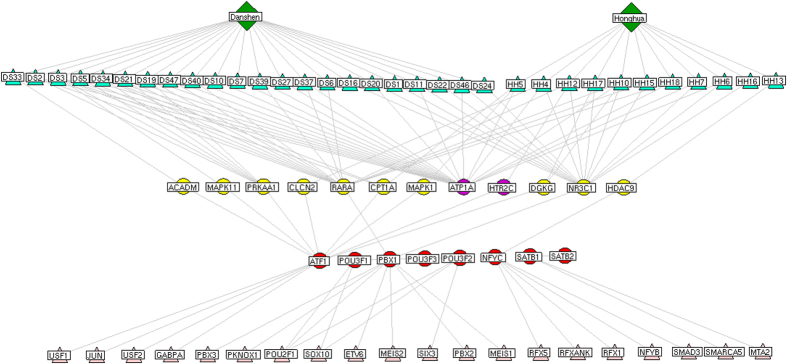
Network of interactions among chemical components of *Radix Salviae miltiorrhizae* (Chinese name DanShen, DS) and *Flos Carthami tinctorii* (Chinese name HongHua, HH), the corresponding putative targets, known therapeutic targets for stroke, eight major TFs and other differentially expressed TFs. Red round nodes refer to the eight major TFs; Yellow refer to the putative targets of DHI; Purple refer to known therapeutic targets for stroke/the putative targets of DHI; Pink refer to other differentially expressed TFs; Green refer to the chemical components of *Radix Salviae miltiorrhizae* and *Flos Carthami tinctorii*.

Finally, the chemical components-major putative targets-major TFs network, which contains 74 nodes, including 2 herbs (*Radix Salviae miltiorrhizae* and *Flos Carthami tinctorii*), 33 chemical components, 8 major TFs, 10 major putative targets of DHI, 2 known therapeutic targets, 27 other differentially expressed TFs and 145 edges, was constructed and shown in Fig. 7. We found that there 22 and 11 chemical components of *Radix Salviae miltiorrhizae* and *Flos Carthami tinctorii*, respectively, had interactions with PBX1, NFYC and ATF1 through their corresponding putative targets, suggesting that these three major TFs might be more closely associated with the therapeutic effects of DHI acting on ischemic stroke.

## Conclusions

In this study, a comprehensive approach integrating a network pharmacology strategy and a newly developed catTFREs method for systematic investigation of target TFs critical to drug-mediated protection against cerebral ischemia was first reported. The high-throughput nature and depth of coverage, as well as the high quantitative accuracy, of the developed approach make it more suitable for analyzing multi-component and multi-target agents. Herein, the approach was successfully applied to analyze the effects of Danhong injection (DHI), a conventional drug for coronary heart disease and cerebral ischemia. After changes in TF activation during the ischemic brain injury in mouse MCAO models were measured using the catTFREs method and an accurate quantitative approach, the target TFs that were critical to DHI-mediated protection against cerebral ischemia were computationally predicted by the network pharmacology strategy. Then, by combining the evaluation of the pharmacological effects of DHI on MCAO mice, the predicted target TFs were experimentally validated. The experimental results indicated that PBX1 and ATF1, along with six other TFs, are putative target TFs for DHI-mediated protection against cerebral ischemia. This study provides the first systematic insight into the target TFs critical to DHI-mediated protection against cerebral ischemia and reveals novel potential therapeutic targets for ischemic stroke.

## Additional Information

**How to cite this article**: Wei, J. *et al*. Systematic investigation of transcription factors critical in the protection against cerebral ischemia by Danhong injection. *Sci. Rep.* **6**, 29823; doi: 10.1038/srep29823 (2016).

## Supplementary Material

Supporting Information

Supplementary Dataset 1

Supplementary Dataset 2

Supplementary Dataset 3

Supplementary Dataset 4

Supplementary Dataset 5

Supplementary Dataset 6
