# Supplementary material for: Systematic investigation of transcription factors critical in the protection against cerebral ischemia by Danhong injection
Source: Sci Rep. 2016 Jul 19;6:29823. doi: 10.1038/srep29823 (PMC4949467; doi:10.1038/srep29823)
Supplement: Supporting Information [file srep29823-s1.doc]

Supporting Information

**Title of Manuscript:** Systematic investigation of transcription factors critical in the protection against cerebral ischemia by Danhong injection

**Author List:** Junying Wei, Yanqiong Zhang, Qiang Jia, Mingwei Liu, Defeng Li, Yi Zhang, Lei Song, Yanzhen Hu, Minghua Xian, Hongjun Yang, Chen Ding, Luqi Huang

Below is the Supporting Information of “Systematic investigation of transcription factors critical in the protection against cerebral ischemia by Danhong injection (Manuscript ID: SREP-15-26556)”.

**Supplementary methods:**

1. Selection of the optimal dosage of Danhong injection (DHI) on ischemic stroke

To find the optimal dosage of DHI on ischemic stroke, high-dosage (10 mL/kg/time), medium-dosage (5 mL/kg/time) and low-dosage (2.5 mL/kg/time) were tested, which are equivalent to the clinical dosage of 9 times, 4.5 times, 3 times, respectively. Experimental results showed that there is no significant difference in blood pressure after administrating MCAO mice with different dosages of DHI (Fig. S2a). Compared with the control group, the high dose administration group was somewhat higher than that in the control group, and the other groups, especially the medium dose group, were relatively stable. Compared with the control group, Longa’s Neurological Severity Score of MCAO mice with different dosages of DHI overall declined (Fig. S2b). The high dose group was not obvious, and the low dose groups decreased significantly, especially in the medium dose group was the most prominent, and has a significant difference (P < 0.01). Compared with the control group, the volume of cerebral infarction in each group was decreased, and there were significant differences among the groups (Fig. S2c). Infarction rate of medium dose group was the most prominent (P < 0.01), and the infarct size was relatively stable. Thus, medium-dosage of DHI (5 mL/kg/time) has the best effect on ischemic stroke. So we selected it in our study.

1. Selection of the loading amount of nuclear protein for EMSA experiments

To find the optimal loading amount of nuclear protein for EMSA experiments, 3 μg, 6 μg, and 12 μg of nuclear extracts of the mouse brain were tested respectively. As shown in Fig. S3, 6 μg of nuclear protein is the optimal choice for EMSA experiments.

**Supplementary figures and table:
Figure S1.** Evaluation of the ischemic injury after MCAO. (a) Longa’s Neurological Severity Score; (b) TTC staining of the brains


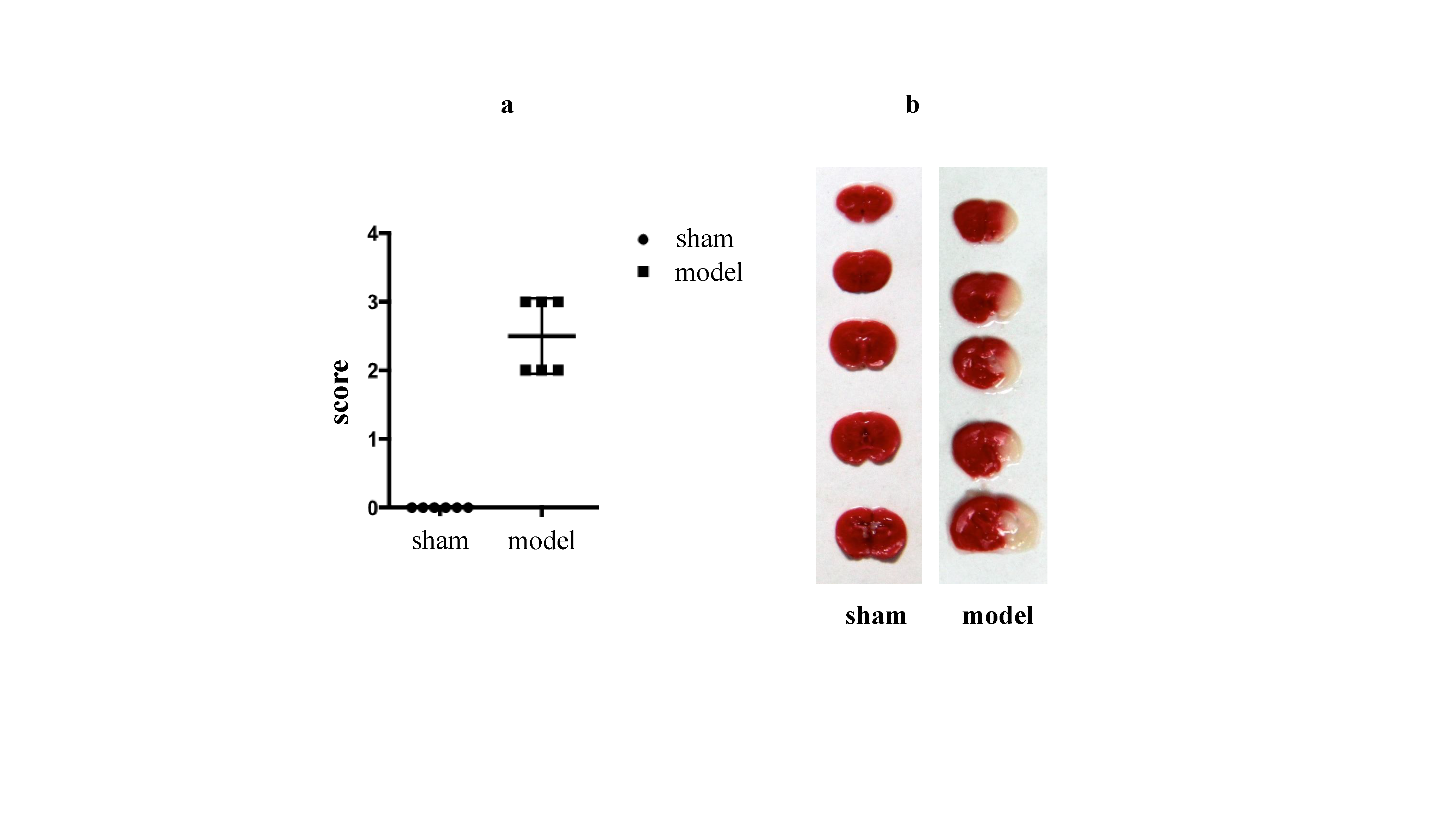


**Figure** **S2**. Selection of the optimal dosage of DHI on ischemic stroke


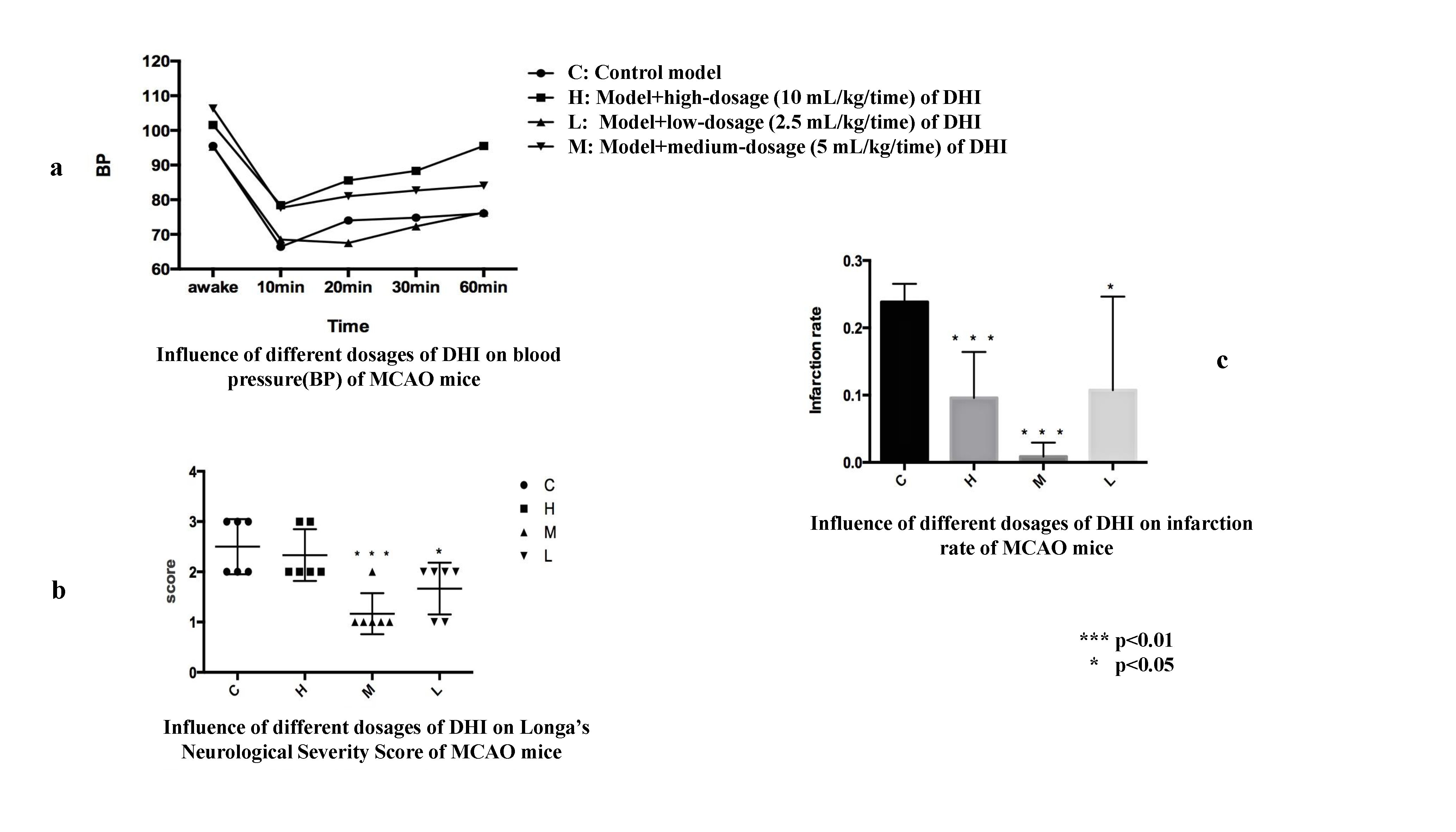


**Figure** **S3**. Selection of the loading amount of nuclear protein for EMSA experiments


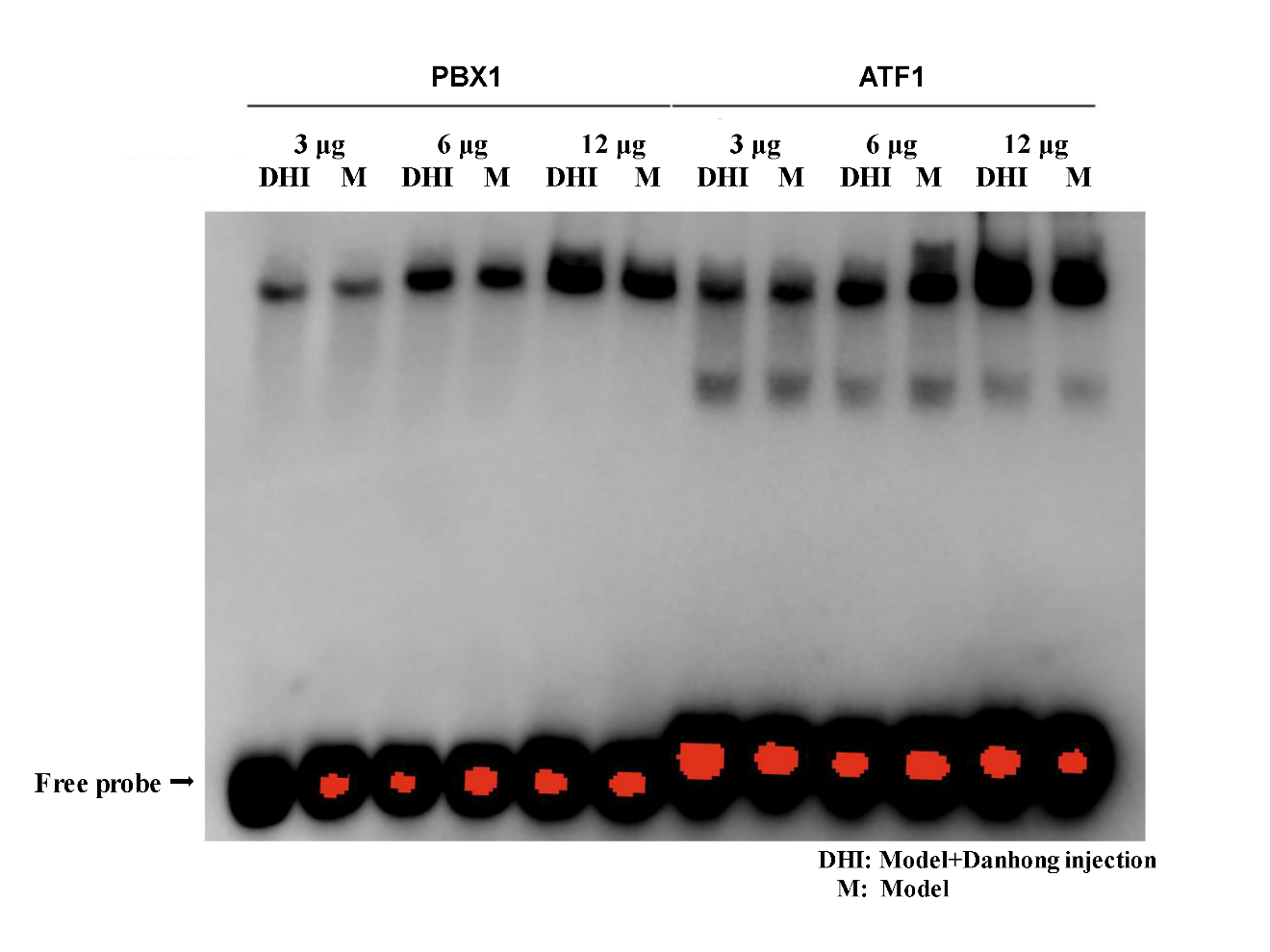


**Table S1** Primers for qPCR

| Gene symbol | Forward primers | Reverse primers |
| --- | --- | --- |
| PF4 | CCCGAAGAAAGCGATGGAGAT | CTTCAGGGTGGCTATGAGCTG |
| HSP70 | GGGAGGACTTCGACAACCG | CTCTTGGCCCTCTCACACG |
| NQO1 | AGTCCATTCCAGCTGACAACC | ACTCCTTTTCCCATCCTCGTG |
| GST | TGACCTGGCAAGGTTACGAAG | AGTTTCATCCCGTCGATCTCTA |
| GAPDH | GCCCAGCAAGGATACTGAGA | GGTATTCGAGAGAAGGGAGGG |
